# Advances and perspectives for antimicrobial peptide and combinatory therapies

**DOI:** 10.3389/fbioe.2022.1051456

**Published:** 2022-12-12

**Authors:** Santos C, Rodrigues G. R, Lima L. F, dos Reis M.C.G, Cunha N.B, Dias S.C, Franco O. L

**Affiliations:** ^1^ S-Inova Biotech, Programa de Pós-Graduação Em Biotecnologia, Universidade Católica Dom Bosco (UCDB), Campo Grande, Brazil; ^2^ Centro de Análises Proteômicas e Bioquímica (CAPB), Programa de Pós-Graduação Em Ciências Genômicas e Biotecnologia, Universidade Católica de Brasília (UCB), Brasília, Brazil; ^3^ Faculdade de Agronomia e Medicina Veterinária (FAV), Universidade de Brasília (UnB), Brasília, Brazil; ^4^ Programa de Pós-Graduação Em Biologia Animal, Universidade de Brasília (UnB), Brasília, Brazil; ^5^ Programa de Pós-Graduação Em Patologia Molecular, Universidade de Brasília (UnB), Brasília, Brazil

**Keywords:** infection medication, antibiotic-therapy combination, antimicrobial resistance, synergic effect, infectious diseases

## Abstract

Antimicrobial peptides (AMPs) have shown cell membrane-directed mechanisms of action. This specificity can be effective against infectious agents that have acquired resistance to conventional drugs. The AMPs’ membrane-specificity and their great potential to combat resistant microbes has brought hope to the medical/therapeutic scene. The high death rate worldwide due to antimicrobial resistance (AMR) has pushed forward the search for new molecules and product developments, mainly antibiotics. In the current scenario, other strategies including the association of two or more drugs have contributed to the treatment of difficult-to-treat infectious diseases, above all, those caused by bacteria. In this context, the synergistic action of AMPs associated with current antibiotic therapy can bring important results for the production of new and effective drugs to overcome AMR. This review presents the advances obtained in the last 5 years in medical/antibiotic therapy, with the use of products based on AMPs, as well as perspectives on the potentialized effects of current drugs combined with AMPs for the treatment of bacterial infectious diseases.

## 1 Introduction

Over the years, antibiotic therapy has been the main tool for treating infectious diseases. Discoveries revealed that antibiotics have been used since ancient civilizations, and it is believed that antibiotics have been in human treatment since 350–550 CE (Iskandar et al., 2022). Since the first discovered classes, it is possible to affirm that antibiotics have been responsible for saving thousands of lives annually since the early 20th century (Yin et al., 2021). Even so, the emergence of difficult-to-treat infectious disease was inevitable, given the co-evolution of pathogens. Selective pressure by antibiotic use has resulted in infectious diseases that are effectively untreatable with conventional antibiotics due to antimicrobial resistance (AMR). An increasing range of AMR organisms including bacteria, viruses, parasites and fungi have caused increased mortality rates around the world (Asokan and Vanitha, 2018). Because of this, AMR has increased considerably worldwide in the hospital environment and has alarmed world health authorities (World Health Organization, 2019). The selective pressure for antibiotic use can be considered mainly responsible for the emergence and increase in AMR (Larsson and Flach, 2022). The fight against multidrug-resistant (MDR) agents is a complex problem in both developed and developing countries (Majumder et al., 2020; Ting et al., 2020; Murray et al., 2022).

In fact, AMR has primarily been associated with selective pressure due to the increased use of antibiotics, and to their indiscriminate use due to self-medication. In addition, AMR can be related to social problems such as world overpopulation, poor sanitation, and invasion of preserved biomes. The use of antibiotics in the agricultural sector and also in the treatment of animals in veterinary clinics has contributed to AMR, as reviewed in (Rather et al., 2017; Aslam et al., 2018). Annual reports associate AMR with high mortality rates, prolonged hospital admission and, consequently, high costs in the healthcare sector. The increase in AMR limits treatment options, as it reduces the possibility of choosing effective drugs that have been used in clinical practice for years (Dadgostar, 2019; World Health Organization, 2020). It is estimated that by 2050 approximately 10 million deaths will be caused by infections caused by AMR (World Health Organization, 2017; Ting et al., 2020).

In a recent report (Global Antimicrobial Resistance and Use Surveillance System (GLASS) Report: Early implementation 2020) (World Health Organization, 2020), the WHO issued an emergency alert on the need to develop new treatment alternatives. Among clinical isolates, bacteria stand out in antimicrobial resistant groups. It is increasingly common to note the inefficiency of traditional antibiotics due to AMR bacteria. In a systematic review published in The Lancet journal, the authors observed that in 2019 alone, among the reports of deaths, about 1.27 million (95% of analyzed reports) were caused by bacteria infections (Murray et al., 2022). Besides stressing the urgency of developing new treatments, 12 representatives of some bacterial families were added to the priority pathogen list by the WHO (Table 1). According to the WHO, those families pose a great threat to humanity (Asokan and Vanitha, 2018).

**TABLE 1:** The bacteria (*n* = 12) considered the greatest threat to human health that were added to the priority pathogen list by the WHO.

Priority Category	Pathogens	Drug-resistant
1 CRITICAL	*Acinetobacter* baumannii	Carbapenem
	*Pseudomonas aeruginosa*	Carbapenem
	Enterobacteriaceae	Carbapenem; ESBL-producing
2 HIGH	*Enterococcus faecium*	Vancomycin
	*Staphylococcus aureus*	Methicillin, vancomycin-intermediate and resistant
	*Helicobacter pylori*	Clarithromycin
	*Campylobacter spp.*	Fluoroquinolone
	*Salmonellae*	Fluoroquinolone
	*Neisseria gonorrhoeae*	Cephalosporin and fluoroquinolone-resist
3 MEDIUM	*Streptococcus pneumoniae*	Penicillin-non-susceptible
	*Haemophilus influenzae*	Ampicillin
	*Shigella spp.*	Fluoroquinolone

Adapted from (World Health Organization, 2017).

Despite the need for new therapeutic options, and regardless of the increase in antimicrobial resistance, it is possible to observe a decline in the development of new drugs and/or discovery of new molecules. There has been no discovery of a new antibiotic class since the “golden era”, the period between the 1950s and 1970s, when the main known classes were discovered (Hutchings et al., 2019). However, this decline is mainly due to the lack of interest from governments and large industries in investing in research. Certainly, evaluating cost-benefit, heavy investments are needed to prospect, obtain and commercialize new drugs. This results in low financial returns in the face of rapidly increasing microbial resistance, even with the high rate of deaths. In addition to the high costs, long periods are generally required for the production and marketing of a new drug. To put this in perspective, it takes 10–15 years for a new product to be approved and made available to the market, with costs of approximately 2 billion dollars (Wouters et al., 2020).

Thus, it is urgent to search for new alternatives that overcome the therapeutic failures of traditional antibiotics. These alternatives include combined therapy or efficient molecule development that is capable of overcoming the increasing wave of threats from resistant organisms. Following the urgency of developing new antimicrobial drugs, many studies have reported proofs-of-concept, indicating the high therapeutic potential of defense peptides, and some peptides are already in clinical trials (Ting et al., 2020). Peptides are also able to boost the effects of other antibiotics, showing an improved action with these drugs. Among these peptides are known antimicrobial peptides (AMPs). AMPs could also be considered host defense peptides (HDR). HDRs have been involved in the living organisms defense system for millions of years (Mishra et al., 2017).

These peptides besides modulating the immune system, was reported that, on *in vivo* models, it can also neutralize endotoxins (Mishra et al., 2017; Boto et al., 2018). HDR also act against pathogens invasion (e.g., viruses, fungi, bacteria, and parasites), and can prevent biofilm formation (Mishra et al., 2017; Boto et al., 2018). In addition, it is possible to find AMPs as anticancer agents (Franco, 2011; Mishra et al., 2017). Additionally, AMPs also can be used for medical products development such as, drugs to be applied in wound healing as well as skin care use, due to antioxidant peptides action (Golonka et al., 2021; Moretta et al., 2021). AMPs can also act as inhibitors of ACE1 (angiotensin converting enzyme 1) and pancreatic lipase, being useful to control metabolic syndrome (Moretta et al., 2021).

As mentioned, those peptides have broad activity spectrum, and although it is possible (Maron et al., 2022), AMP resistance is not prevalent. It is an advantage compared to conventional drugs, and AMPs can act synergistically with conventional antibiotics potentializing the effects of them (Duong et al., 2021; Zhu et al., 2022). In this context, the synergistic action of AMPs associated with current antibiotic therapy can bring important results for the production of effective new drugs to overcome AMR. This review presents the advances obtained over the last 5 years in the medical/antibiotic therapy scene, with the use of products based on AMPs, as well as perspectives on the synergic effect of AMPs in association with current drugs for the treatment of bacterial infectious diseases.

## 2 Synergic effect of drug combination therapy used in clinical practice

For decades, antibiotics have facilitated the treatment of bacterial infectious diseases. However, over the years, selective pressure, by antibiotics use, allowed that bacteria to acquire a random change in their DNA. With those changes, the bacteria can survive in the antibiotic’s presence. This way, bacteria developed resistance mechanisms to conventional antibiotics (Ghosh et al., 2020). Also, those mechanisms can be horizontally transferred. Basic horizontal gene transfer (HGT) mechanisms were discovered more than 50 years ago. In general, HGT is an important form of bacterial survival and evolution (Ting et al., 2020; Power et al., 2021). In this way, horizontal transfer can occur with resistance genes. Resistance gene are also transferred between pathogenic and non-pathogenic bacteria including antibiotic resistance (ABR) ones. HGT afford pathogenic bacteria to develop resistance by obtaining ABR genes by exchange between other pathogenic ones, or commensal bacteria present in surrounding environments (Ting et al., 2020). However, antimicrobial misuse can cause resistance and effect the efficacy of drugs (Boto et al., 2018).

The therapeutic potential of existing antibiotics through collaborations with other biological or chemical molecules represents an approach to enhance antibacterial activity and inhibit the emergence of resistance (Shang et al., 2019). Combined action between two or more drugs that contribute to the final result against a multi-resistant microorganism is known as synergism. Synergistic interaction involves lower doses of the combination constituents, allowing a therapeutic effect (Lloyd et al., 2020). The enhanced effect of therapeutical molecules reflects an efficient means of increasing the amplitude of cellular responses induced by stimulation levels, like AMPs, for example (Sechet et al., 2018; Duong et al., 2021).

Drug–drug interactions (DDIs) happen when one drug modifies another’s pharmaceutical activity, but these require multiple dose levels in the analysis of each drug, either alone or in different combination ratios (Niu et al., 2019). Intuitively, molecule combination may represent higher effectiveness. However, drugs that could be combined, commonly did not improved the collateral effects if it not has dosage reduction of each molecule present in the combination (García-Fuente et al., 2018). DDIs are significant since metabolic biotransformations arise at some point between drug absorption into the circulation and its elimination (Katzung and Trevor, 2012). The biotransformation process can leave this xenobiotic in bioactivation through changes in elimination rates. Therefore, synergistic molecular combinations can overcome toxicity and the effects associated with high doses of drugs, allowing the dosage of each compound to be reduced or accessing a specific target (Roemhild et al., 2022).

Valuable examples of combinatorial effects were described in tests using colistin associated with 19 antibiotics against 20 colistin-resistant and 15 carbapenem-resistant Enterobacteriaceae isolates. The method used in this work was inkjet printer-assisted digital dispensing checkerboard array (Brennan-Krohn et al., 2018). Eighteen of nineteen combinations demonstrated synergy against two or more isolates. In addition, four higher synergic combinations (colistin combined with linezolid, rifampin, azithromycin, and fusidic acid) show potentialized effects against ≥90% of strains. These results suggest that colistin may exert a sub-inhibitory permeabilizing effects on the Gram-negative bacterial outer membrane, even in isolates that are resistant to it (Brennan-Krohn et al., 2018).

Another *in vitro* study tested the combination of colistin with teicoplanin multidrug-resistant *Acinetobacter* spp (Rady et al., 2022). This combination was tested against 29 multidrug-resistant *Acinetobacter spp* isolates. The researchers used 1 mg l^−1^ colistin and 10 mg l^−1^ teicoplanin in combination and demonstrated *in vitro* synergism against all tested *Acinetobacter* isolates except one (*Acinetobacter lowffii*). This combination also demonstrated a bactericidal effect at 6 h against 100% of *A. baumannii* isolates with no bacterial regrowth at 24 h. The same combination was bactericidal against three out of seven non-baumannii *Acinetobacter* isolates. Colistin can act on *Acinetobacter spp*. Outer membrane and permit teicoplanin to reach its target in the cell wall (Rady et al., 2022).

As explained, the association of two or more antibiotics demonstrates therapeutic potential through association against microorganisms; the production of antibiotics is not fully species-specific. Antibiotics can be composed of organisms associated with different species, genera, or even orders. An antibiotic’s mechanism of action must begin when it identifies the target macromolecule and its function. For example, the susceptibility of different bacteria to antibiotics relies chiefly on the structure of their cell walls, as it completes the capability of the antibiotic to penetrate the bacterial cell (Long, 1951; Liu et al., 2021). However, pathogen resistance is a result of pathogen selective pressure over many years. The molecular modification of the pathogen to cheat the antibiotic mechanism of action makes the pathogen more efficient, making infections even more difficult to treat (Uddin et al., 2021). However, synergic effects of AMPs with other antimicrobials can reduce the risk of developing bacterial resistance, due to the high lethality level (Duong et al., 2021).

## 3 Clinically significant advances in synergism between AMP/antibiotic and AMP/AMP

As mentioned, new therapies are urgently needed to tackle the global problem of AMR. To overcome the therapeutic limitations created by bacterial resistance, some strategies have been considered (Vargas-Casanova et al., 2019). In addition to antibiotic combinations, which are a common practice in the hospital environment, antibiotic association with AMPs has been demonstrated as a potential resource against AMR agents (dos Santos et al., 2021; Li et al., 2021). Recent reports can demonstrate the increased activity of the combination (AMP-antibiotic), revealing a potentialized effect. Table 2 summarizes some proof-of-concept works that have reported synergistic AMP-antibiotic combinations in the last 5 years.

**TABLE 2:** Some reports of *in vitro* studies evaluating the synergistic effect of AMP and commercial antibiotic combination, published in the last 5 years.

Combination	Medical target/disease	Sponsor/collaborators
Peptide SPR741 and antibiotics (rifampin, clarithromycin, and azithromycin)	*E. coli* ATCC 25922*, K. pneumoniae* ATCC 43816*, and A. baumannii* NCTC 12156	Corbett et al. (2017)
Peptides (r(P)ApoBL and r(P)ApoBS) and antibiotics (ciprofloxacin, colistin, erythromycin, kanamycin sulfate, and vancomycin)*	*E. coli* methicillin-resistant *S. aureus* MRSA and ATTC 29213*, Salmonella enteriditis* 706 RIVM*, B. globigii* TNO *BM013, B. licheniformis ATTC 21424, P. aeruginosa* ATCC 27853*, and* PAO1	Gaglione et al. (2017)
Peptides KR-12-a5 and antibiotics (Chloramphenicol, Ciprofloxacin, Oxacillin)	MDR *P. aeruginosa* strains	Kim et al. (2017)
Peptide Nisin Z and Novobiocin	*S. aureus, S. epidermidis, E. coli*	Lewies et al. (2017)
Peptides (Melittin, Nisin Z) and Novobiocin	*S. aureus* ATCC 12228 and 12,600	Lewies et al. (2017)
leftPeptide CLP and antibiotics (ampicillin, ceftazidime, erythromycin, levofloxacin)	*A. baumannii E. coli, P. aeruginosa, S. aureus*	Li et al. (2017)
Peptide MP-AF and antibiotics (Cephalothin, Chloramphenicol, Gentamicin, Neomycin, Ciprofloxacin, Trimethoprim/sulfamethoxazole, SXT)	*E. coli ATCC 25922, PFL6, PFH13 A. baumannii* clinical isolates	Lin et al. (2017)
Peptide hLF1-11 and antibiotics (Clarithromycin, Clindamycin, Gentamycin, Rifampicin, Tigecycline)	*K. pneumoniae* (6 different strains)	Morici et al. (2017)
Peptide SET-M33 and antibiotics (meropenem, rifampin, aztreonam, tobramycin, ciprofloxacin)	MDR *Klebsiella pneumoniae, Pseudomonas aeruginosa, and Acinetobacter baumannii*	Pollini et al. (2017)
Peptide (FK-13-a1 and FK-13-a7) and chloramphenicol	*E. coli, P. aeruginosa, S. typhimurium, B. subtilis, S. epidermidis, S. aureus*	Rajasekaran et al. (2017)
Peptide LL-37 and antibiotics (ceftriaxone, ciprofloxacin)*	*Salmonella enterica* serotype Newport	Sakoulas et al. (2017)
DP7 and antibiotics (vancomycin and azithromycin)	MDR *S. aureus, P. aeruginosa, A. baumannii, and E. coli*	Wu et al. (2017)
Peptide Cecropin A2 and Tetracycline	*P. aeruginosa* PA14, PA103, clinical isolates	Zheng et al. (2017)
Peptide SPR741 and rifampin*	*A. baumannii*	Zurawski et al. (2017)
Peptide Ocellatin-PT3 and antibiotics (Ceftazidime, Ciprofloxacin)/Peptide P5 and Meropenem	*MDR P. aeruginosa Pa1 anda Pa4-SA2/P. aeruginosa*	Bessa et al. (2018)
Peptides (HHC-10, 1018, DJK-5) and antibiotics (Gentamicin, ampicillin, tetracycline, chloramphenicol, spectinomycin)*	MDR Gram-positive and Gram-negative	Pletzer et al. (2018)
Peptide SPR741 and azithromycin	Enterobacteriaceae isolates	Stainton et al. (2018)
Peptide PrAMP and colistin	*K. pneumoniae* and A. baumannii	Otvos Jr et al. (2018)
Peptide A3-APO and colistin	MRD *Klebsiella pneumoniae* K97/09	Otvos Jr et al. (2018)
Peptide A3-APO and imipenem*	MDR *K. pneumoniae Acinetobacter* baumannii BAA-1605	Otvos Jr et al. (2018)
Peptide A3-APO and meropenem	CRU *E. coli*	Otvos Jr et al. (2018)
Peptide melittin and doripenem, doxycycline, colistin	A. baumannii, P. aeruginosa	Akbari et al. (2019)
Peptide SPR741 and azithromycin	Enterobacteriaceae	Akhoundsadegh et al. (2019)
Peptide colistin and antibiotics (chloramphenicol, tetracycline, linezolid, vancomycin)	P. aeruginosa, *E. coli*, A. baumanii, S. marcescens	Armengol et al. (2019)
Peptide SPR741 and piperacillin-tazobactam, ceftazidime, and aztreonam*	Human study NCT03022175/NCT03376529	Eckburg et al. (2019)
Peptide SPR741 and rifampicin	*E. coli*	French et al. (2019)
Peptide CAMPs and antibiotics (colistin, imipenem)	Methicillin-resistant S. aureus and MDR P. aeruginosa.	Geitani et al. (2019)
Melimine, Mel4 and protamine, and antibiotics (cefepime and ciprofloxacin)	S. aureus P. aeruginosa	Kampshoff et al. (2019)
Peptide (DPK-060 and LL-37) and monolaurin lipid nanocapsules	S. aureus biofilms	Rozenbaum et al. (2019)
Peptides (L11W, L12W, and I4WL5W) and antibiotics (ampicillin, ceftazidime, penicillin, erythromycin, tetracycline)	MDR S. aureus	Shang et al. (2019)
Peptide L12 and antibiotics (ceftazidime, erythromycin, gentamycin, levofloxacin, linezolid, oxacillin, tetracycline, vancomycin)	MR S. aureus	Xiong et al. (2019)
Peptide HNP-1 and hBD-1 and cefotaxime*	S. aureus isolates	Bolatchiev et al. (2020)
Peptide G3KL and propidium iodide	*K. pneumoniae*, P. aeruginosa	Gan et al. (2020)
Peptide tridecaptin M and antibiotics (rifampicin, vancomycin, and ceftazidime)	A. baumannii	Jangra et al. (2020)
Peptide cLFchimera and antibiotics (gentamicin, cefazolin, ceftazidime)	*E. coli*, P. eruginosa and S. typhi	Roshanak et al. (2020)
Peptide Pt5-1c snf oxacillin, vancomycin, streptomycin, and azithromycin	MDRs S. aureus USA500, *E. coli* 577, and *K. pneumoniae* 2182	Duan et al. (2021)
Nisin and colistin, P10 and ceftazidime/doripenem	*Acinetobacter* baumannii P. aeruginosa	Jahangiri et al. (2021)
Peptide D-11 and antibiotic vancomycin	Gram-negative pathogens P. aeruginosa	Li et al. (2021)
Peptidomimetic CEP-136 and	*E. coli, K. pneumoniae, A. baumannii, and P. aeruginosa*	Mood et al. (2021)
LL-37 and colistin	*E. coli*	Morroni et al. (2021)
Peptides (S1-Nal and S1-Nal-Nal) and vancomycin	*P. aeruginosa E. coli*	Wu et al. (2021)
Peptides (FK20; FdK; dFdK and LK20) and imipenem	*A. baumannii* biofilms	Caraway et al. (2022)
Peptides (GVF27, FAM-GVF27, LL-37) and ciprofloxacin	Bcc and antibiofilm activity	Bosso et al. (2022)
Peptides (CATH-1, CATH-3, PMAP-36) and erythromycin	*S. aureus, S. enteritidis, E.coli*	Lu et al. (2022)

*Also *in vivo* assay; CRU, Carbapenem-resistant uropathogenic; MDR, multidrug-resistant *Klebsiella pneumoniae* K97/09; Bcc, *Burkholderia cepacia* Complex.

Bacterial species names are italicized.

AMPs can be biological or chemical molecules working with antibiotics. Broadly, two physical features are accepted for AMPs: a cationic charge and a significant measurement of hydrophobic residues. The first property promotes selectivity for negatively charged microbial cytoplasmic membranes, and the second property facilitates interactions with the fatty acyl chains (Shen et al., 2018; Cantor et al., 2019). AMPs interacting with bacterial membranes may cause pores leading to an osmotic imbalance and promoting cell disruption (Hollmann et al., 2018). In this way, it can facilitate the entry of other molecules such as conventional antibiotics and further affect different targets within the cell (Hollmann et al., 2018) such as, cell wall biosynthesis, mechanism of protein synthesis, interference of metabolic activity and synthesis and integrity of nucleic acids (Sarkar et al., 2017; Lin et al., 2018; Stokes et al., 2019; Lade and Kim, 2021).

Combination therapy consist in a strategy to overcome bacterial resistance to conventional antibiotics, as well as, enhance the molecules efficiency (Wang et al., 2022). This is because some AMPs-antibiotics combination can allow bacterial pores to open longer preventing pore repair, and further increasing cellular osmolarity unbalance. In addition, it can complement bacterial killing by imparting mechanisms other including the reduction of bacterial resistance and host cell toxicity (Duong et al., 2021). Bacterial resistance reduction could be considered in AMP-antibiotic combination, once that this synergistic combination involves multiple targets. In general, those targets are in independent bacteria cell pathways. Therefore, to overcome the mechanism of action to both molecules, present in the combination, also would be necessary multiples independents, and simultaneous, set of mutations, in the bacteria for it to become resistant (Duong et al., 2021; Zhu et al., 2022). Thus, the AMP-antibiotic combination can boost antibacterial effects as well as interrupting biofilm formation, and can act with more efficiency than individual drugs (Wu et al., 2017).

Given that AMPs have dominant activities against multidrug-resistant organisms, they can be used to treat the increasing number of antibiotic-resistant infections (Giuliani et al., 2007). It is known that membrane disarray is the principal mechanism of AMPs that can kill or inhibit microorganisms (Atefyekta, 2020), since the notable targets of AMPs are the cell membranes of microbes. The mechanism of action for AMPs has been extensively studied in recent years, and readers are directed to three reviews that detail this mechanism (Moravej et al., 2018; Raheem and Straus, 2019; Benfield and Henriques, 2020).

Other studies demonstrated the action of synergic combinations between AMPs and antibiotics. The combination of colistin sulfate-tobramycin was tested to kill *Pseudomonas aeruginosa* biofilms. *In vivo* tests were conducted by groups of 10 female Lewis rats (age, 7 weeks) who were challenged intratracheally with 0.1 ml of a suspension of alginate beads containing 1 x 10^8^ cfu/mL *P. aeruginosa* PAO1 in the left lung. After one hour, the rats received 0.1 ml of the colistimethate or tobramycin or a simultaneous combination of both drugs or 0.9% saline *via* intratracheal. After 7 days, the rats who received the antibiotic combination therapy significantly reduced the number of *P. aeruginosa* cells. In addition, the authors also did a pilot study with 5 patients who presented cystic fibrosis. They inhaled colistin and then tobramycin for 4 weeks, and the results demonstrated a reduction of 2.52 ± 2.5 log10 cfu of *P. aeruginosa* per milliliter of sputum (*p* = 0.027) (Herrmann et al., 2010).

The human neutrophil peptide (HNP)-1 was used in combination with isoniazid and rifampicin against *Mycobacterium tuberculosis*. Results *in vitro* demonstrated the reduction of >1-log unit of *M tuberculosis* even when HNP-1 and anti-TB drugs were used at 1/16 MICs. The *in vivo* analyses demonstrated that the use of HNP-1 in conjunction with anti-TB drugs resulted in a significant decrease in bacterial load in the lungs, liver and spleen of the infected animals, compared with control animals (Kalita et al., 2004).

Another study evaluated the potentialized effect in cryptdin 2 (a Paneth cell antimicrobial peptide) and ampicillin (Amp) combination against *Salmonella enterica* serovar Typhimurium. For *in vivo* tests, mice were infected with 10^7^ CFU of *S. typhimurium* orally. After 7 days the mice were separated into 11 groups of five mice each and treated with cryptdin 2 and ampicillin. Cryptdin 2 was injected subcutaneously (s.c.) at a dose of 5 μg/mouse, while Amp was administered s. c. at 16, 32, and 64 mg/kg of body weight, individually and in combination. Results suggest larger log unit decreases in all target organs of mice treated with the combination than those for the drugs used alone. According to these studies, the synergic effect of AMPs with other antimicrobials are responsible for the efficient control or killing of bacteria. (Duong et al., 2021).

As cited, some works have reported promising results with the antibiotic-AMP or AMP-AMP associations, and these have been proven to be a potent antimicrobial therapy (Grassi et al., 2017; Sierra and Viñas, 2021). Furthermore, the AMP-AMP combination also can display stronger effect. The expectation of potentialized antimicrobial effect in AMPs combination seems likely to master the problem of resistance to conventional antibiotics, being the AMPs potential candidates against multi-drug resistant bacteria (Pirtskhalava et al., 2021). In the last 5 years some studies have explored the synergistic effects of combining two or more AMPs in the control of MDR (Table 3).

**TABLE 3:** Some reports evaluating the synergistic effect of AMP-AMP combination, published in the last 5 years.

AMP combination	Medical target/disease	Authors
Coleoptericin and defensin	*S. aureus*	Zanchi et al. (2017)
PGLa and magainin 2	*E. coli* and *S. aureus*	Zerweck et al. (2017)
A3-APO and K97/09	*K. pneumoniae*	Otvos Jr et al. (2018)
LL-37: CAMA: magainin-II: nisin^a^	*Methicillin-resistant S.aureus* and MDR *P. aeruginosa*	Geitani et al. (2019)
Diptericins and attacins	*Providencia burhodogranariea*	Hanson et al. (2019)
Lysostaphin and LL-37	*S. aureus*	Sadeghi et al. (2019)
HNP-1 and hBD-1	*S. aureus* isolates	Bolatchiev et al. (2020)
PGLa and Magainin 2	*E. coli and S. aureus*	Bechinger et al. (2020)
VG16KRKP and KYE28	*Xanthomonas, Pseudomonas*	Ilyas et al. (2019)
PGLa and magainin 2	*E. coli*	(Glattard et al., 2016; Zerweck et al., 2017; Ma et al., 2020)
Magainin 2 and tachyplesin 1	*E. coli*	Remington et al. (2020)
Ib-AMP4 and E50-52	MDR *Staphylococcus aureus*	Satei et al. (2021)
zopfiellasins A–D	*P. syringae pv. actinidiae*	Zhang et al. (2021a)
ε-PL and nisin	*S. aureus and E. coli*	Gao et al. (2022)
Peptide mixture	Gram-positive and negative strains	Santos et al. (2022)
RPM cocktail (FK20; FdK; dFdK and LK20)	*A. baumannii* biofilms	Caraway et al. (2022)

^a^ Also *in vivo* assay; RPM, random peptide mixtures.

The combination of two or more AMPs is a strategy that can act on multiple targets, in different pathogens. Also, it looks like the peptide mixture might be safe, as demonstrate a work that evaluated the peptide pool. Peptides mixture can also besides to being considered to improve antimicrobial and antibiofilm activity, *in vitro* bioassay also was demonstrated that the mixture can be safe to human tissue (Santos et al., 2022). Random peptide mixture (RPM) has been an alternative to overcoming to antimicrobial treatment potentialization. Same RPM can attenuate mouse mortality when submitted to sepsis infection (Caraway et al., 2022). RPM consist in performed synthetic peptide by incorporation of a defined proportion of two or more amino acids, making RPM extreme diversity. So, it tourn improbably the rapid bacterial resistance (Bauer et al., 2020; Bennett et al., 2021). In other study was evaluated the RPM cocktail effect (dFDK, LK20, FK20, and FdK peptides) *in vitro*, and demonstrated that four RPM (5 µg ml^−1^ each of RPM) in a cocktail (20 µg ml^−1^) were more effective against all 3 *A. baumannii* isolates tested than single RPM (100 µg ml^−1^), even the single RPM having been the able to capable to eradicate *A. baumannii* biofilms and inhibited mouse models of infection (Caraway et al., 2022). The results *in vitro* also However, the single RPM was capable to eradicated *A. baumannii* biofilms (Bennett et al., 2021; Caraway et al., 2022). In addition to increasing the lethality, this may be more efficient from the point of view of the variety of pathogens that a single combination can control (Geitani et al., 2019; Hanson et al., 2019; Bechinger et al., 2020).

Combination therapy with AMPs can be an alternative to reduce cytotoxicity by synergism. Those strategies not only can surpass the bactericide effect of conventional antibiotics but also can decrease the dose of bactericide necessary, and decrease toxicity rates and side effects of individual or combined antibiotics (Duong et al., 2021; Sierra and Viñas, 2021). AMP-antibiotic and AMP-AMP combinations have the objective of boosting the effects of traditional antibiotics that are still in use. They can also boost the action of antibiotics that have lost their efficacy over the years due to selective pressure from resistant bacteria. The improved effect of the AMP-antibiotic association can mitigate the potential mechanism of bacterial resistance. However, clinical trials involving AMP-antibiotic or AMP-AMP interaction are still scarce. Synergism studies and clinical trials urgently need to get the health agencies’ approval and rapidly obtains products to be marketed. AMP-antibiotic or AMP-AMP combination strategies may bolster the fight against difficult-to-treat infections (Zhang Q.-Y. et al., 2021).

## 4 Concluding remarks

The increase in the number of deaths worldwide, caused by infections that are difficult to treat, has caused serious concern among world health authorities. The WHO has been warning for some time about the need to develop new drugs. The spread of AMR is not a new event, but even so, there is still a lack of tools for adequate treatment (Luong et al., 2022). Since the golden age of antibiotics, there have been no reports of new molecules, and it is urgently necessary to discover new classes of antimicrobial agents capable of combating infections caused by MDR organisms. Antimicrobial peptides have shown great potential, emerging as a new source of bioactive compounds, capable of combating MDR agents. AMPs have been extensively studied, and over the years some have already been accepted for clinical trials and others have already been used in some therapies. It is known that AMPs promote low toxicity and, due to the high specificity of these molecules, the development of resistance to AMPs is rare, a fact that confirms that AMPs are a powerful weapon in the fight against AMRs. (Luong et al., 2022).

Even so, co-evolution of the pathogen, as observed with conventional antibiotics, can also occur with AMPs, so it is needed to be prepared. The selective pressure that has led many bacteria to resist conventional antibiotics can also be observed using AMP therapies. Although resistance to AMPs is rarer, some isolates have shown such resistance. The combination therapy strategy, with two or more antibiotics, is common in clinical practice, but the risks and benefits have to be weighed carefully (Fatsis-Kavalopoulos et al., 2022). Antibiotic combination has been successful, but there are already clinical isolates that proved to be multidrug resistant. In addition to conventional combination therapy (among antibiotics), positive effects of AMPs-antibiotics combination have been reported. The main advantages of this combination can reduce the annual costs caused by infections and can also prevent emergence of AMR agents (Wang et al., 2022).

This AMP-antibiotic combination has attracted attention due to the benefits it may have. Several studies have been reported in recent years with promising results from AMP-antibiotic combinations. The various proof-of-concept studies with this combination have standardized methods. They have also facilitated the determination of the mechanism of action and synergism mechanisms in AMP combinations. (Pizzolato-Cezar et al., 2019).

However, despite the results demonstrating the positive action of AMP-antibiotic combinations, some challenges are still faced, especially in relation to the permeability/stability of AMPs (Pizzolato-Cezar et al., 2019). Stabilization of AMPs is still the main challenge in clinical therapy. Although there is no direct solution, some work involving nanostructures for the delivery of these molecules has drawn attention in the search to stabilize AMPs and ensure interaction with the target. Nanostructures can be a reinforcement in drug delivery, including for AMPs (Mercer et al., 2020; Yang et al., 2021).

Several AMPs have been discovered with pharmaceutical potential, showing promise for the development of alternative antimicrobial drugs. They have provided support for the management of AMR, including the possibility of AMP-AMP combinations. Studies involving the combination reveal that AMPs act synergistically with other AMPs, with conserved characteristics, due to the high specificity of these molecules. The higher the specificity, the lower the doses needed to obtain results. AMPs can be used in combination with antibiotics and other AMPs and exhibit strong antimicrobial effects even at low concentrations. The dose-effect relation also reflects on the adverse effects caused by the therapy. The fewer doses needed, the more limited the unwanted side effects. Besides the low doses required, another advantage of the increased effect of the AMP-antibiotic or AMP-AMP combination is the reduction of production costs and hospital costs with patient interaction. New drugs will be developed in the future, based on proofs of concept that demonstrate the great potential of AMPs and their combinations (Duong et al., 2021).
